# SorLA in Interleukin-6 Signaling and Turnover

**DOI:** 10.1128/MCB.00641-16

**Published:** 2017-05-16

**Authors:** Jakob Vejby Larsen, Claus Munck Petersen

**Affiliations:** The MIND Center, Department of Biomedicine, Aarhus University, Aarhus, Denmark

**Keywords:** IL-6, IL-6R, STAT3 signaling, SorLA, Vps10p domain receptors, cytokines, endocytosis

## Abstract

Interleukin-6 (IL-6) is a multifunctional cytokine with important functions in various physiologic processes. Mice lacking IL-6 exhibit multiple phenotypic abnormalities, such as an inadequate immune and acute-phase response, and elevated levels of circulating IL-6 have been found to accompany several pathological conditions. IL-6 binds the nonsignaling IL-6 receptor (IL-6R), which is expressed as a transmembrane, as well as a secreted circulating protein, before it engages homodimeric gp130 for signaling. Complex formation between IL-6 and the membrane-bound IL-6 receptor gives rise to classic *cis* signaling, whereas complex formation between IL-6 and the soluble IL-6R results in *trans* signaling. Here, we report that the endocytic receptor SorLA targets IL-6 and IL-6R. We present evidence that SorLA mediates efficient cellular uptake of both IL-6 and the circulating IL-6R in astrocytes. We further show that SorLA interacts with the membrane-bound IL-6R at the cell surface and thereby downregulates IL-6 *cis* signaling. Finally, we find that the SorLA ectodomain, released from the cell membrane upon enzymatic cleavage of full-length SorLA, may act as an IL-6 carrier protein that stabilizes IL-6 and its capacity for *trans* signaling.

## INTRODUCTION

Interleukin-6 (IL-6) is a member of the human four-helical bundle IL-6 cytokine family, which also includes the heterodimeric cardiotrophin-like cytokine:cytokine-like factor 1 (CLC:CLF-1), ciliary neurotrophic factor (CNTF), leukemia inhibitory factor (LIF), cardiotrophin-1, oncostatin M, IL-11, IL-27, and IL-31 (1). It is a 21- to 28-kDa glycosylated protein that is secreted by immunocompetent cells like macrophages, T cells, and B cells, as well as by nonimmune cells such as adipocytes, muscle cells, and neurons (2), and it is important in a diversity of physiologic processes implicating, e.g., hepatocytes, cells of the nervous system, hematopoietic progenitor cells, and mononuclear lymphocytes (2–6). Accordingly, IL-6 knockout (ko) mice exhibit several phenotypic abnormalities, including an inadequate immune response to certain pathogens and an impaired hepatic acute-phase response (7). IL-6 engages homodimeric gp130 for signaling via the janus kinase (JAK) and the signal transducer and activator of transcription 3 (STAT3) pathway, but in order to do so it must first complex with its primary receptor which exists as a full-length (nonsignaling), transmembrane protein (IL-6R), as well as a soluble secreted receptor (sIL-6R) consisting only of the IL-6R ectodomain. Complex formation between IL-6 and either sIL-6R or transmembrane IL-6R gives rise to *trans* and classic *cis* signaling, respectively. Expression of full-length IL-6R, and *cis* signaling, seems to be restricted to relatively few cell types, notably hepatocytes, lymphocytes, and microglia (2). In contrast, sIL-6R, resulting from proteolytic cleavage and shedding of the IL-6R ectodomain and/or from cellular secretion of an alternative splice variant of the receptor (8–11), is found in circulation, and as gp130 is ubiquitously expressed, almost all cell types have potential for *trans* signaling (12–14). It is therefore not surprising that many *in vivo* functions of IL-6 can be blocked by specific inhibition of the *trans* signaling pathway (15).

Since they are both prerequisites for signaling, deficiency in either IL-6 or IL-6R result in very similar phenotypes characterized by an impaired immune response and a defective acute-phase response (7, 16). Elevated levels of circulating IL-6 and sIL-6R, on the other hand, frequently accompany pathophysiologic conditions and constitute potential drug targets in the treatment of diseases like rheumatoid arthritis, asthma, and multiple sclerosis (3, 12, 17–19).

We show here that the endocytic receptor SorLA may impact the cellular uptake as well as the signaling of IL-6. Human SorLA is one of the five type 1 receptors that constitute the human Vps10p-domain (Vps10p-D) receptor family (20, 21). It is expressed in most regions of the nervous system but is also found in a number of nonneuronal tissues and cell types such as the liver, kidney, pancreas, and cells of the immune system, e.g., monocytes and macrophages (20, 22–24). Apart from an N-terminal Vps10p-D comprising a unique ligand-binding ten-bladed β-propeller supported by two minor domains (25, 26), the luminal part of SorLA also contains a small β-propeller domain with an associated epidermal growth factor class B-like motif and a cluster of 11 low-density lipoprotein receptor class A repeats (21). SorLA's cytoplasmic tail is short but contains several motifs for the binding of adaptor proteins, such as AP-1 and -2, GGA1 to -3, and the retromer complex, that are involved in endocytosis, basolateral sorting, and trafficking between Golgi and endosomal compartments (27–32).

The extracellular part of SorLA binds a broad spectrum of ligands encompassing platelet-derived growth factor, neuropeptides such as neurotensin (NT), neurotrophic factors, elements of the plasminogen activator system, proteins involved in lipoprotein metabolism, and cytokines (31, 33–37), as well as proteins anchored in the cell membrane, e.g., amyloid precursor protein (APP), glia cell line-derived neurotrophic factor (GDNF) family receptor α1 (GFRα1), and the CNTF receptor (CNTFR) (33, 34, 38). Both the Vps10p-D and the class A repeats are ligand binding, but importantly, the former carries a propeptide that prevents binding until it is removed by enzymatic cleavage in late Golgi compartments (35, 37).

In agreement with the above findings, SorLA displays a variety of different functions. It affects APP processing and Aβ amyloid generation (38), and mutations in SorLA constitute a risk factor for familiar and sporadic forms of Alzheimer's disease (39). It is further implicated in the cellular release of lipoprotein lipase (LPL) (31) and is linked to obesity and glucose tolerance (40, 41). It also influences receptor tyrosine kinase RET signaling via interaction with GDNF and its primary receptor GFRα1 (34). Furthermore, SorLA is subject to cleavage and cellular shedding of its luminal part (sSorLA) (42), and sSorLA in circulation is implicated in the migration of smooth muscle cells and may serve as a biomarker for atherosclerosis, coronary stenosis, and diabetic retinopathy (43–47). Likewise, elevated levels of sSorLA in cerebrospinal fluid are a potential marker of progressive Alzheimer's disease (48).

We have recently demonstrated that SorLA targets the heterodimeric cytokine CLC:CLF-1 (via the CLF-1 subunit) and its primary receptor, the CNTFR, and that it modulates the cellular response to CLC:CLF-1 by mediating CLF-1-dependent endocytosis and downregulation of the CNTFR (33). In the present study, we show that SorLA targets IL-6 and the IL-6R, and we investigate the functional implications of these interactions in cells. Our study reveals that full-length SorLA conveys the cellular uptake of both IL-6 and sIL-6R (individually or in complex) and also interacts with the membrane-bound IL-6R blocking its binding of IL-6. The findings further suggest that, whereas full-length SorLA may downregulate IL-6 *cis* signaling, sSorLA may stabilize IL-6 and its capacity for *trans* signaling.

## RESULTS

### SorLA binds IL-6 and mediates its cellular uptake.

**FIG 1:**
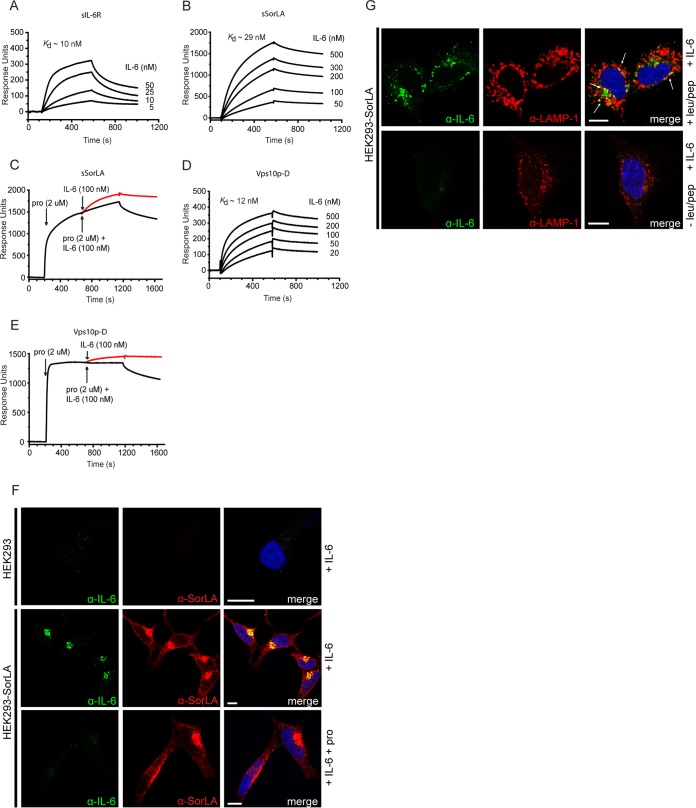
Binding (SPR analysis) of IL-6 to sIL-6R, sSorLA, and the Vps10p-D of SorLA (A to E) and SorLA-mediated uptake of IL-6 in cells (F) and in LAMP-1-positive vesicles (G). The concentration dependence of IL-6 binding to immobilized sIL-6R (A), sSorLA (B), and SorLA Vps10p-D (D) was evaluated. IL-6 was applied at the given concentrations, and the indicated *K_d_* values were calculated on the basis of the collected sum of data. SorLA propeptide-mediated inhibition of IL-6 (100 nM) binding to sSorLA (C) and the SorLA Vps10p-D (E). SorLA was subjected to saturating concentrations of propeptide (2 μM) prior to injection of a mixture of propeptide (2 μM) and IL-6 (100 nM). The projected red lines indicate IL-6 binding obtained in the absence of propeptide, i.e., curves to be expected if the propeptide does not inhibit. (F) Untransfected and SorLA-transfected HEK293 cells were incubated (37°C, 30 min) at 125 nM IL-6 in the absence or presence of 20 μM SorLA propeptide (pro) as indicated. Subsequently, the cells were washed, fixed, permeabilized, and stained using goat anti-IL-6 and mouse anti-SorLA as primary antibodies, as well as Alexa Fluor 488-conjugated donkey anti-goat and Alexa Fluor 568-conjugated donkey anti-rabbit antibodies as secondary antibodies. (G) Immunofluorescence showing accumulation and LAMP-1 colocalization of IL-6 (arrows) in cells treated with lysosomal inhibitors prior to and during 3 h of incubation with IL-6 (Pearson's *r* = 0.42). Scale bars, 10 μm.

Initially, we examined the binding of IL-6 to the immobilized ectodomains of IL-6R and SorLA (sIL-6R and sSorLA) using surface plasmon resonance (SPR) analysis. As demonstrated in Fig. 1A and B, IL-6 exhibited concentration dependent high-affinity binding not only to sIL-6R (*K_d_* ∼ 10 nM) but also to sSorLA (*K_d_* ∼ 29 nM). The binding to sSorLA was completely inhibited in the presence of a surplus of SorLA's own propeptide (Fig. 1C) but was almost unchanged by neurotensin (NT) (data not shown). IL-6 also bound to the purified SorLA Vps10p-D (*K_d_* ∼ 12 nM) (Fig. 1D) in competition with the SorLA propeptide (Fig. 1E).

We next studied the binding of IL-6 to full-length SorLA in cells. Wild-type (wt) HEK293 cells and cells stably transfected with SorLA were incubated at 125 nM IL-6 (30 min, 37°C) in medium supplemented or not supplemented with 20 μM SorLA propeptide or NT. The cells were fixed, permeabilized, and stained with anti-IL-6 and anti-SorLA antibodies. As shown (Fig. 1F), staining was undetectable in wt cells, whereas cells transfected with SorLA displayed a significant intracellular staining for both proteins and an almost complete colocalization of IL-6 with SorLA (Pearson's *r* = 0.74 ± 0.04 [mean ± standard error of the mean {SEM}, *n* = 9]). The uptake was unchanged at a surplus of 20 μM NT (not shown) but was almost abolished in the presence of a surplus of SorLA propeptide. In cells treated with lysosomal inhibitors (leupeptin and pepstatin A), internalized IL-6 accumulated and to some extent colocalized with LAMP-1, strongly suggesting that SorLA targeted IL-6 for degradation in the lysosomes (Fig. 1G).

**FIG 2:**
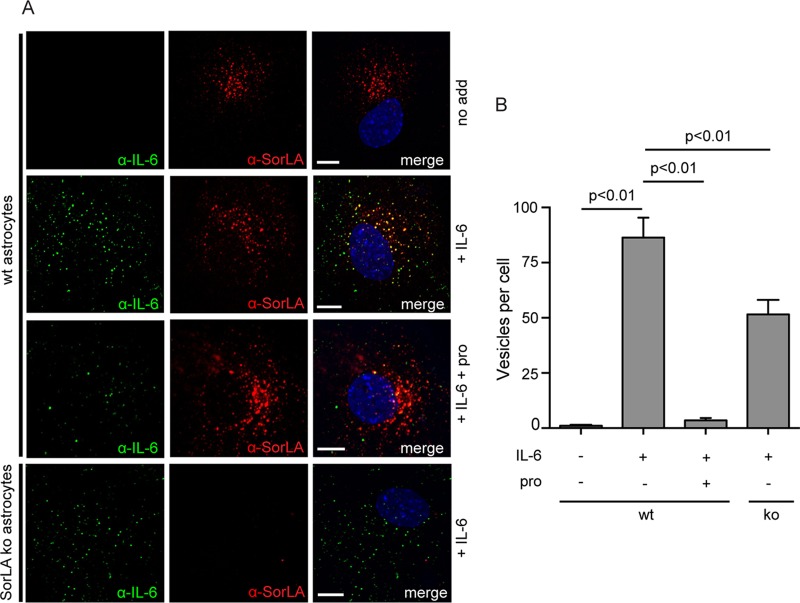
Uptake of IL-6 in astrocytes is inhibited by SorLA propeptide. (A) Astrocytes isolated from wt and SorLA ko mice were incubated (37°C, 30 min) in unsupplemented medium or in medium supplemented with 125 nM IL-6 with or without 20 μM SorLA propeptide. The cells were then washed prior to fixation and permeabilization before staining with goat anti-IL-6 and mouse anti-SorLA antibodies and the proper secondary antibodies. (B) Histogram showing the average number of IL-6 containing vesicles per cell as determined by automated counting in 15 randomly selected astrocytes. Each column represents mean value, and bars indicate the SEM. The data were evaluated using one-way ANOVA, and post hoc analysis was carried out using Tukey's test. Scale bars, 10 μm.

To further substantiate these findings, we examined the uptake of IL-6 in astrocytes, which exhibit endogenous expression of SorLA, as well as sortilin and IL-6R mRNA, but little or no detectable IL-6R protein (49). Cultured astrocytes isolated from wt mice were incubated (30 min, 37°C) with or without 125 nM IL-6 in the absence or presence of 20 μM SorLA propeptide prior to staining with anti-IL-6 and anti-SorLA antibodies. As demonstrated in Fig. 2A, astrocytes incubated in the presence of IL-6 showed a substantial vesicular uptake of IL-6, which to a large extent colocalized with SorLA (Pearson's *r* = 0.47 ± 0.03 [mean ± SEM, *n* = 15]). This uptake was practically abolished in the presence of a surplus of SorLA propeptide. Thus, as determined by automated counting of 15 randomly selected cells, astrocytes incubated at 125 nM IL-6 contained 159 ± 10 (mean ± SEM) positive vesicles per cell, whereas 25 ± 5 positive vesicles were found in cells coincubated with IL-6 and SorLA propeptide and only 6 ± 2 positive vesicles were seen in controls not subjected to IL-6 (Fig. 2B). The results in SorLA ko astrocytes (lower panel in Fig. 2A, far right column in Fig. 2B) demonstrated a comparatively reduced uptake of IL-6 (∼ 41%) in the absence of SorLA, and practically no uptake in the presence of SorLA's propeptide, which, apart from SorLA, also inhibits sortilin. Since sortilin also binds IL-6 (50), the data strongly suggest that SorLA and sortilin account for the uptake of IL-6 in astrocytes. Taken together, these data demonstrate that IL-6 selectively binds the SorLA Vps10p-D and that SorLA conveys the cellular uptake and endocytosis of IL-6.

### Soluble IL-6R targets SorLA and is internalized.

**FIG 3:**
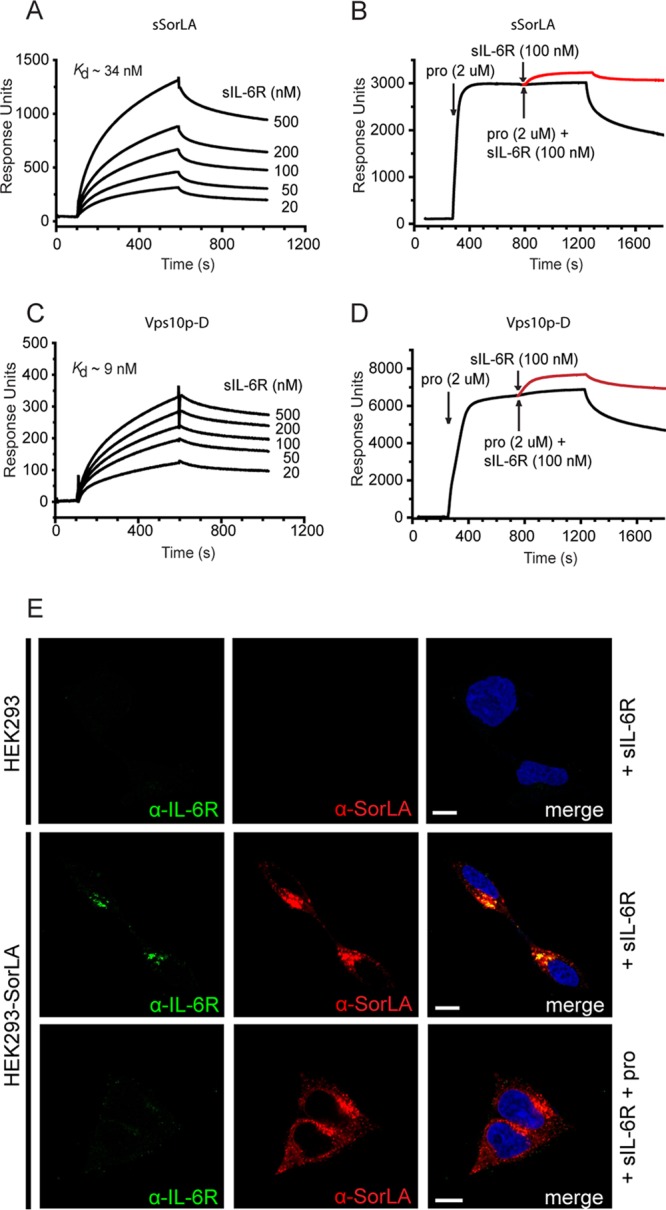
SPR analysis of the binding of sIL-6R to sSorLA and to the Vps10p-D of SorLA (A to D) and SorLA-mediated uptake of sIL-6R in cells (E). The concentration dependence of sIL-6R binding to immobilized sSorLA (A) and the SorLA Vps10p-D (C) was evaluated. The indicated *K_d_* values were calculated on the basis of the collected sums of data. The inhibition by SorLA propeptide (pro) of sIL-6R binding to sSorLA (B) and to the Vps10p-D (D) was also evaluated. SorLA was preincubated with a saturating concentration of propeptide (2 μM) prior to the injection of a mixture of propeptide (2 μM) and sIL-6R (100 nM). The response obtained with sIL-6R alone, i.e., the curve to be expected in the absence of inhibition, is indicated in each case in red. (E) Uptake of sIL-6R in SorLA-transfected and wt HEK293 cells. The cells were incubated (37°C, 30 min) at 250 nM sIL-6R in the absence or presence of 20 μM SorLA propeptide. The cells were then washed, fixed, permeabilized, and subsequently stained with mouse anti-IL-6R and rabbit anti-SorLA as primary antibodies and with Alexa Fluor 488-conjugated goat anti-mouse and Alexa Fluor 568-conjugated goat anti-rabbit antibodies as secondary antibodies. Scale bars, 10 μm.

Using a similar setup, we next sought to determine whether SorLA also interacts with sIL-6R. SPR analysis demonstrated (Fig. 3A to D) that it does and that sIL-6R displays much the same binding characteristics as IL-6. Thus, sIL-6R bound SorLA with high affinity (*K_d_* ∼ 34 nM) (Fig. 3A), and the binding was completely inhibited by the SorLA propeptide (Fig. 3B), but only partly (∼ 50%) by NT (not shown). This suggested binding to the Vps10p-D, and, in agreement, the purified Vps10p-D was found to bind sIL-6R with an affinity (*K_d_* ∼ 9 nM) comparable to that of the entire extracellular part of SorLA (Fig. 3C) and, as before, binding was completely inhibited in the presence of the SorLA propeptide (Fig. 3D).

The interaction between sIL-6R and full-length SorLA was confirmed in wt and SorLA-transfected HEK293 cells. The cells were incubated (30 min, 37°C) at 250 nM sIL-6R and subsequently stained with anti-IL-6R and anti-SorLA antibodies. As apparent from Fig. 3E, the SorLA transfectants, unlike wt HEK293, displayed a heavy intracellular sIL-6R stain colocalizing with SorLA (Pearson's *r* = 0.72 ± 0.03 [mean ± SEM, *n* = 17]), signifying a considerable SorLA mediated uptake of the soluble receptor. In accordance with the SPR analysis, coincubation with the SorLA propeptide completely blocked the cellular uptake of sIL-6R.

**FIG 4:**
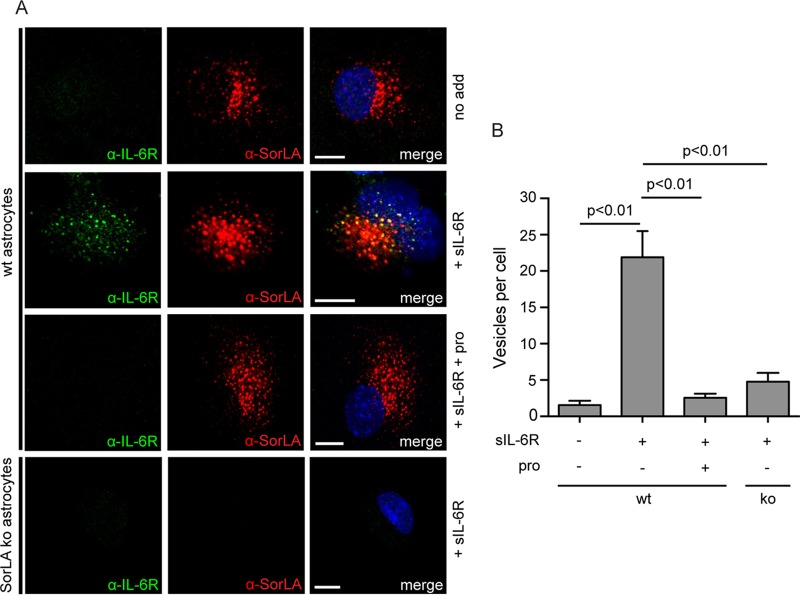
SorLA accounts for the uptake of sIL-6R in astrocytes. (A) Astrocytes isolated from wt and SorLA ko mice were incubated (37°C, 30 min) in unsupplemented medium or in medium containing 250 nM sIL-6R with or without 20 μM SorLA propeptide (pro) as indicated. The cells were washed, fixed, and permeabilized before staining with mouse anti-IL-6R, rabbit anti-SorLA antibodies, and the matching secondary antibodies. (B) Histogram showing the average number of sIL-6R positive vesicles found in each of nine randomly selected wt and SorLA ko astrocytes. Each column represents the mean value, and bars indicate the SEM. The data were evaluated using one-way ANOVA and Tukey's test. Scale bars, 10 μm.

The uptake of sIL-6R was finally examined in cultured astrocytes isolated from wt and SorLA ko mice. It appears (Fig. 4A) that whereas SorLA-deficient cells did not present any internalization of sIL-6R, wt astrocytes exhibited a significant vesicular uptake largely colocalizing with SorLA (Pearson's *r* = 0.40 ± 0.06 [mean ± SEM, *n* = 9]). Again, this uptake was minimized in the presence of a surplus of SorLA propeptide. Quantification of the sIL-6R containing vesicles revealed that SorLA ko cells—and cells not exposed to sIL-6R—contained 5 ± 1 and 2 ± 1 (means ± SEM, *n* = 9) sIL-6R-positive vesicles per cell, respectively, as opposed to wt astrocytes that contained 25 ± 4 positive vesicles per cell upon incubation with sIL-6R and as little as 3 ± 1 in cultures supplemented with SorLA propeptide (Fig. 4B). It follows that sIL-6R, similarly to IL-6, targets the SorLA Vps10p-D and is internalized by SorLA in cells.

### Membrane-bound IL-6R and SorLA form a complex on the cell surface.

**FIG 5:**
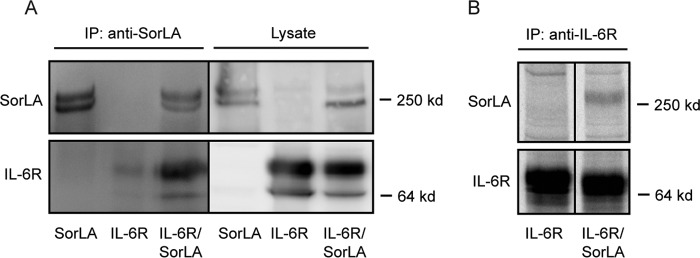
Interaction between full-length IL-6R and SorLA in cells. (A) HEK293 cells transfected with SorLA or IL-6R or both receptors in combination were incubated with the non-membrane-permeable chemical cross-linker DTSSP (2 nM). After 45 min, the reaction was stopped, and the receptor proteins were immunoprecipitated from cell lysates with anti-SorLA. Reduced samples of precipitate were analyzed by SDS-PAGE and Western blotting. (B) HEK293 cells transfected with IL-6R or IL-6R/SorLA were biolabeled (4 h, 37°C) using [^35^S]cysteine and [^35^S]methionine and then washed and lysed. Subsequently, receptor proteins were immunoprecipitated from cell lysates using anti-IL-6R and analyzed by reducing SDS-PAGE and autoradiography.

To determine whether SorLA also targets the full-length transmembrane IL-6R, cross-linking experiments were performed on transfected HEK293 cells. Transfectants expressing either IL-6R, SorLA, or both in combination were treated with the thiol-cleavable and membrane-impermeable chemical cross-linker DTSSP. After 45 min, the reaction was stopped, the cells were lysed, and cross-linked adducts were subsequently immunoprecipitated (anti-SorLA Ig) and subjected to reducing SDS-PAGE and immunoblot analysis with anti-SorLA and anti-IL-6R Ig. As apparent from Fig. 5A, IL-6R was undetectable (not precipitated) in SorLA single transfectants, and only a weak band resulted from IL-6R transfectants, whereas a comparatively much stronger band was produced by adducts precipitated from IL-6R/SorLA double transfectants. Coprecipitation was also seen in the absence of cross-linker (Fig. 5B). The data evidently indicate that membrane-bound IL-6R and SorLA, when coexpressed, interact on the cell surface membrane.

### Functional implications of the SorLA–IL-6R interaction.

**FIG 6:**
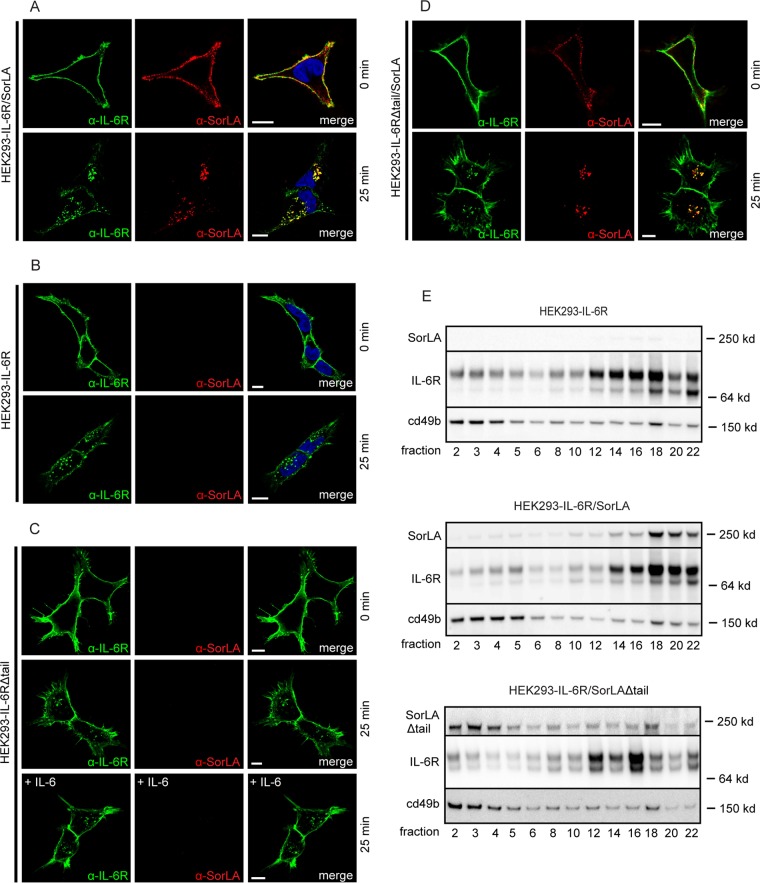
Influence of SorLA and the cytoplasmic IL-6R domain on the internalization and localization of IL-6R in cells. HEK293 transfectants expressing IL-6R (B) or IL-6RΔtail alone (C) or coexpressed with SorLA (A and D) were incubated (4°C, 2 h) with mouse anti-IL6R and rabbit anti-SorLA, washed, and then reincubated at zero time in warm media (37°C) supplemented or not supplemented with 25 nM IL-6. At the indicated times (0 and 25 min), the cells were fixed, permeabilized, and stained with Alexa Fluor 488-conjugated goat anti-mouse and Alexa Fluor 568-conjugated goat anti-rabbit antibodies. (E) HEK293 cells transfected with IL-6R, IL-6R/SorLA, or IL-6R/SorLAΔtail were subjected to subcellular fractionation. The fractions were subjected to Western blotting and probed with antibodies against SorLA, IL-6R, and cd49b (control). Scale bars, 10 μm.

The findings discussed above suggest that SorLA may impact the functions of the full-length transmembrane IL-6R. Since IL-6R early on was reported to internalize poorly on its own (51, 52), we speculated whether SorLA could facilitate its endocytosis and trafficking. To that end, HEK293 cells, either IL-6R single transfectants or IL-6R/SorLA double transfectants, were incubated with anti-IL-6R and anti-SorLA antibodies (2 h, 4°C). The cells were then washed and reincubated in unsupplemented warm (37°C) medium, followed by fixation either immediately (zero time) or after 25 min, and eventually stained using matching secondary antibodies. As shown in Fig. 6A, double-transfected cells presented a distinct surface staining for both IL-6R and SorLA at zero time, and at 25 min the staining was predominantly intracellular, displaying a significant degree of IL-6R and SorLA colocalization (Pearson's *r* = 0.78 ± 0.02 [mean ± SEM, *n* = 22]), indicating a simultaneous rapid and efficient internalization of both receptors. Notably, however, internalization of full-length IL-6R appeared equally effective in IL-6R single transfectants (Fig. 6B), whereas an IL-6R construct without the cytoplasmic tail (IL-6RΔtail) presented only a minor degree of endocytosis (Fig. 6C), even upon coexpression with SorLA (Fig. 6D) or in the presence of 25 nM IL-6 (Fig. 6C), which has been reported to facilitate rapid gp130-mediated downregulation of IL-6R (53). Thus, IL-6R has the capacity to internalize on its own, and this capacity relies on its cytoplasmic domain and is unaffected by SorLA and ligand binding.

Subcellular fractionation of HEK293 transfectants further indicated that SorLA has little or no influence on the cellular localization and trafficking of IL-6R in general. Thus, the subcellular distribution of IL-6R (Fig. 6E, top panel) was virtually unchanged upon coexpression with SorLA (middle panel), as well as upon coexpression with a SorLA construct lacking the cytoplasmic tail (SorLAΔtail) the subcellular localization of which differs distinctly from that of wt SorLA (bottom panel).

**FIG 7:**
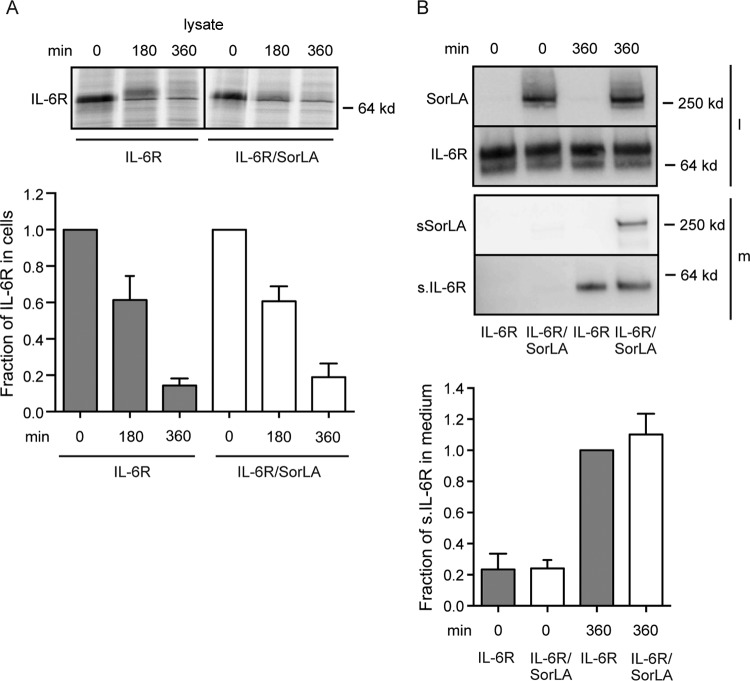
Cellular half-life and shedding of IL-6R upon coexpression with SorLA. (A, upper panel) HEK293 cells expressing IL-6R or IL-6R/SorLA were biolabeled (4 h, 37°C) with [^35^S]cysteine and [^35^S]methionine in the presence of brefeldin A, washed, and incubated in warm medium (37°C). At the indicated time points, IL-6R was immunoprecipitated from cell lysates and analyzed by reducing SDS-PAGE and autoradiography. The lower panel (histograms) shows quantification of band densities (means ± SEM) in three similar experiments. Results are given relative to results obtained at zero time (assigned the value 1). (B) IL-6R and SorLA in medium and cell lysates of HEK293 single and double transfectants. The cells were incubated in culture medium for 0 or 360 min, and the content of IL-6R and SorLA found in the medium (m) and in the cell lysate (l) was detected by Western blotting and quantified by using densitometry of the specific bands. The histogram (B, lower panel) shows the estimated amounts of shed IL-6R (found in the medium) relative to the amount of IL-6R found in the medium of IL-6R single transfectants at 360 min of incubation. Each column represent mean values, and bars indicate the SEM (*n* = 5).

Additional experiments were set up to determine whether SorLA influences the cellular turnover/half-life of the IL-6R. IL-6R single transfectants and IL-6R/SorLA double transfectants were biolabeled prior to washing and reincubation in unsupplemented medium at zero time. At given time points (0, 180, and 360 min), IL-6R was immunoprecipitated from cell lysates and analyzed by reducing SDS-PAGE and autoradiography. The result (Fig. 7A) shows similar half-lives of IL-6R in the two cell lines. Likewise, Western blot detection of unlabeled receptors released to the medium in a corresponding experiment (Fig. 7B) demonstrated that the cellular release of IL-6R was similar in single and double transfectants.

It can be concluded from the above that although IL-6R and SorLA appear to interact on the cell membrane, SorLA has no major impact on the sorting, endocytosis, or shedding of IL-6R. Thus, the findings are in good agreement with previous reports demonstrating that basolateral sorting of IL-6R in polarized cells is mediated by the IL-6R cytoplasmic domain and that monoclonal antibodies decrease the cellular uptake of IL-6 by blocking its binding to the receptor (54, 55).

### SorLA downregulates IL-6 *cis* signaling.

**FIG 8:**
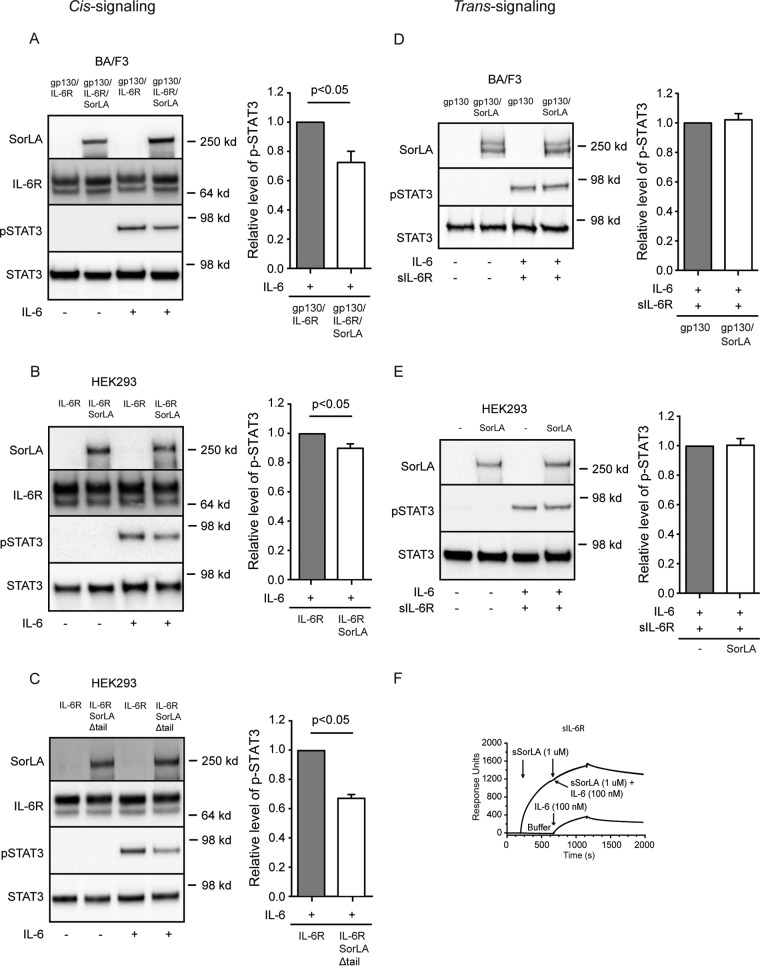
IL-6 *cis* signaling but not *trans* signaling is affected in SorLA transfectants. (A) BA/F3 cells transfected with gp130 and IL-6R alone or in combination with SorLA were incubated (37°C, 15 min) in the absence or presence of 5 nM IL-6. The levels of total and phosphorylated STAT3 were subsequently determined by Western blotting and densitometry. (B and C) HEK293 cells, with endogenous expression of gp130, and transfected with IL-6R alone or in combination with SorLA (B) or SorLAΔtail (C) were stimulated with IL-6 and probed for STAT3 and pSTAT3 as described above. (D) BA/F3 transfected with gp130 or double transfected with gp130 and SorLA were incubated (37°C, 15 min) in blank medium or medium containing both IL-6 and sIL-6R (5 nM each). The cell lysates were analyzed for STAT3 and pSTAT3 as described above. (E) Wild-type HEK293 expressing gp130 and corresponding SorLA transfectants were stimulated and analyzed as in panel D. The left panels (A to E) show Western blot results of single experiments; the right panels sum up the results of several experiments and show results obtained in SorLA transfectants (open columns) relative to values obtained in cells not transfected with SorLA (shaded columns). Each column represents the mean values, and bars indicate the SEM (A, *n* = 9; B, *n* = 6; C, *n* = 6; D, *n* = 4; E, *n* = 7). *P* values were calculated using a Wilcoxon signed-rank test based on raw data. (F) SPR analysis of the binding of IL-6 to immobilized sIL-6R in the presence of a surplus of sSorLA. Soluble IL-6R was subjected to sSorLA (1 μM) prior to the injection of a mixture of sSorLA (1 μM) and IL-6 (100 nM). The response obtained with IL-6 (100 nM) alone is shown.

Since signaling is another function that may be affected by complex formation between SorLA and IL-6R on the cell membrane, we next assessed the phosphorylation of STAT3 (pSTAT3) upon IL-6 *cis* and *trans* signaling in BA/F3 and HEK293 cells. IL-6 *cis* signaling was initially tested in BA/F3 cells, which has no endogenous expression of gp130, IL-6R, or SorLA. Cells transfected with either gp130/IL-6R or gp130/IL-6R/SorLA were incubated with or without 5 nM IL-6 for 15 min, and their response in terms of pSTAT3 was subsequently determined by Western blotting. As apparent from Fig. 8A, cells expressing SorLA showed a significantly lower level (∼28%) of pSTAT3 than cells without SorLA. Similar experiments were then performed in HEK293 cells, which have a minor endogenous expression of both gp130 and SorLA. The cells were transfected with either IL-6R, IL-6R/SorLA, or IL-6R/SorLAΔtail and stimulated with 5 nM IL-6 as described above. In agreement with the result in BA/F3 cells, HEK293 cells coexpressing IL-6R and SorLA presented a lower (∼10%) content of pSTAT3 than the IL-6R single transfectants (Fig. 8B), and an even lower level (∼ 33%) was seen in cells transfected with SorLAΔtail which, in contrast to wt SorLA, accumulates on the surface membrane (Fig. 8C). In both cases the difference was significant (*P* < 0.05), as determined by Wilcoxon signed-rank test. In contrast, transfection with SorLA in BA/F3 and HEK293 cells (not expressing IL-6R) did not significantly alter the response to stimulation with a combination of IL-6 and sIL-6R (Fig. 8D and E). An SPR analysis was finally performed to determine whether SorLA might inhibits the binding of IL-6 to the IL-6R. As shown in Fig. 8F, the binding of IL-6 was completely abolished when IL-6R had been subjected to a saturating concentration of sSorLA.

Taken together the results suggest that expression of SorLA downregulates IL-6 *cis* signaling, presumably by interacting with the membrane-bound IL-6R and thereby hampering formation of the signaling complex by blocking the association between IL-6 and the membrane-bound IL-6R. On the other hand, SorLA does not affect IL-6 *trans* signaling, which involves the soluble form of the IL-6R.

### Soluble SorLA may stabilize IL-6 and its capacity for *trans* signaling.

**FIG 9:**
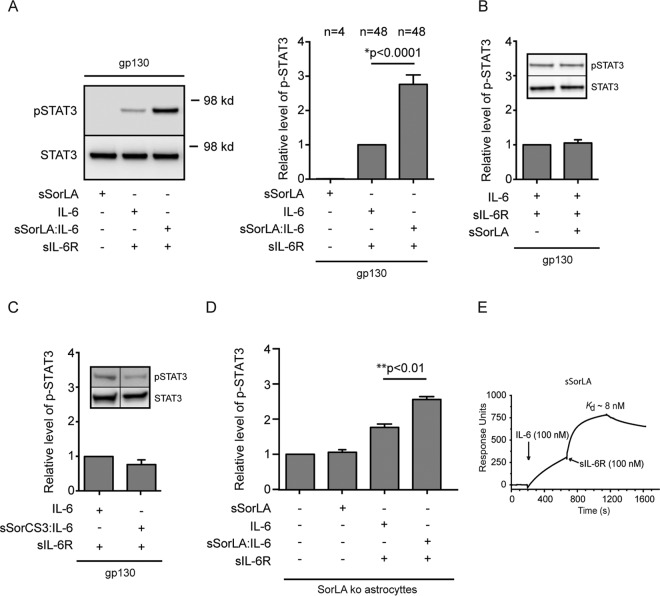
Soluble SorLA may stabilize IL-6 *trans* signaling. (A) BA/F3 cells expressing gp130 were stimulated (15 min, 37°C) as indicated, and the resulting levels of pSTAT3/STAT3 were determined by Western blotting of cell lysates. Prior to stimulation, sIL-6R (5 nM) and sSorLA (40 nM) were incubated separately (3 h, room temperature), whereas IL-6 (5 M) was preincubated alone or in combination with 40 nM sSorLA (sSorLA:IL-6). The left panel shows a Western blot of a representative experiment. The right panel summarizes results of several (*n*) experiments in which the pSTAT3 levels (measured by densitometry) were set relative to the level obtained in response to IL-6+IL-6R (assigned the value 1). Bars indicate the SEM, The *P* value was calculated using the Wilcoxon signed-rank test. (B) The same BA/F3 cells were stimulated (15 min, 37°C) as indicated, but in this case none of the reagents had been coincubated prior to stimulation. The relative pSTAT3 levels were determined as described above. The inset depicts a Western blot of a single experiment, and the histogram summarizes results of nine experiments. (C) pSTAT3 levels in the same cells and stimulated as for panel A except that sSorLA had been substituted with sSorCS3. The inset shows a Western blot of a single experiment, the histogram summarizes (as described above) results of five separate experiments. (D) pSTAT3 levels in SorLA ko astrocytes stimulated with preincubated reagents as for panel A. The columns represent mean values (± SEM, *n* = 3) relative to the pSTAT3 level in unstimulated astrocytes (assigned value 1). Data were evaluated by using one-way ANOVA and Tukey's test. (E) SPR analysis of the binding of sIL-6R to a preformed sSorLA:IL-6 complex. Immobilized sSorLA was initially exposed to IL-6 (100 nM) prior to the injection of fresh buffer containing 100 nM sIL-6R. The subsequent increase in response units signifies the binding of sIL-6R to the preformed sSorLA:IL-6 complex. The estimated *K_d_* is indicated.

As described previously (42) and demonstrated in Fig. 7B SorLA exhibits TACE mediated shedding of its luminal part at the cell surface whereby a circulating soluble receptor (sSorLA) is generated. To examine whether sSorLA may complex with IL-6 and affect IL-6 *trans* signaling, sSorLA and IL-6 were coincubated for 3 h at room temperature to allow them to form a complex (sSorLA:IL-6). In parallel, sSorLA, IL-6, and sIL-6R were each incubated separately under similar conditions. BA/F3 cells expressing gp130 were then stimulated for 15 min with either IL-6 and sIL-6R (5 nM each), sSorLA:IL-6 (40 nM:5 nM) and sIL-6R (5 nM), or just sSorLA (40 nM). The cells were subsequently lysed and the level of pSTAT3 determined by Western blotting. As evident from Fig. 9A, cells subjected exclusively to sSorLA did not result in any phosphorylation of STAT3, whereas cells stimulated with IL-6 and sIL-6R (*trans* signaling) gave rise to an increment in pSTAT3. Interestingly, sSorLA:IL-6 in combination with sIL-6R enhanced the observed IL-6 *trans* signaling by almost 3-fold. In contrast, if sSorLA and IL-6 were not allowed to coincubate prior to cell stimulation, the presence of SorLA did not increase IL-6 *trans* signaling (Fig. 9B), indicating that sSorLA must complex with IL-6 in order to affect signaling. Moreover, IL-6 preincubated with the luminal part of SorCS3 (sSorCS3), a SorLA-related receptor with a 10-fold lower affinity for IL-6 (not shown), did not result in enhanced IL-6 *trans* signaling (Fig. 9C). To confirm the above findings, similar experiments were performed using SorLA ko astrocytes, as astrocytes do not express the membrane-bound IL-6R but respond to IL-6 *trans* signaling (13, 49). As evident from Fig. 9D, sSorLA in it self had no effect, but upon coincubation and complex formation with IL-6, it significantly increases IL-6 *trans* signaling. It follows from these findings that sSorLA may complex with IL-6 and positively affect IL-6 *trans* signaling.

To elaborate on the mechanism underlying the above finding, we performed an additional SPR analysis, in which immobilized sSorLA was preexposed to IL-6 prior to sIL-6R binding. As shown in Fig. 9E, sIL-6R displayed a significant binding to the preformed sSorLA:IL-6 complex, and the *K_d_* was found to be 8 ± 2 nM (mean ± SEM, *n* = 3), which is similar to the *K_d_* of ∼10 nM measured between IL-6 and sIL-6R (Fig. 1A). Thus, these data strongly suggest that sIL-6R is able to bind sSorLA-bound IL-6 and give rise to a trimeric sSorLA:IL-6:sIL-6R complex. Since sSorLA was required to coincubate (complex) with IL-6 in order to affect signaling and, furthermore, did not seem to modify the affinity between IL-6 and sIL-6R, we speculated that sSorLA might stabilize circulating IL-6 and thereby preserve its capacity for signaling. In order to explore this possibility, HEK293 cells transfected with IL-6R were stimulated (15 min) with 5 nM preincubated (3 h at room temperature) IL-6 or with 5 nM IL-6 not subjected to preincubation. The level of pSTAT3 was then determined by Western blotting, revealing that preincubated, compared to nonpreincubated, IL-6 resulted in a 27% ± 2% lower level of pSTAT3 (mean ± SEM, *n* = 9; *P* < 0.01 [Wilcoxon signed-rank test]), i.e., preincubation had reduced the capacity of IL-6 for signaling. When preincubated in the presence of sSorLA, however, IL-6 appeared to preserve its activity (Fig. 9A and D). These findings strongly suggest that sSorLA may serve as a stabilizing carrier protein for extracellular and circulating IL-6 and thereby maintain its functional half-life and *trans* signaling capability.

## DISCUSSION

It is well known that IL-6 binds the IL-6R and subsequently recruits homodimeric gp130 for signaling. We report here that the sorting receptor SorLA binds IL-6, as well as the transmembrane and the soluble forms of the IL-6R. Upon binding, SorLA mediates efficient cellular uptake and internalization of both IL-6 and sIL-6R. SorLA does not affect the internalization or cellular localization of IL-6R; it does, however, hamper its capacity for IL-6 binding and for the induction of IL-6 *cis* signaling. In contrast, our findings show that the (shed) ectodomain of SorLA may act as an IL-6 carrier protein that stabilizes circulating IL-6 and thus preserves its capacity for *trans* signaling.

### SorLA mediates uptake and internalization of IL-6 and sIL-6R.

IL-6 and sIL-6R display similar binding patterns to SorLA and to the isolated Vps10p-D. Both bind with high affinity and in both cases binding are completely abolished by SorLA's propeptide. Since the propeptide is known to prevent ligand binding to the Vps10p-D (37), without preventing the binding of ligands (e.g., LPL) that target alternative sites in the luminal part of SorLA (31, 37), our data demonstrate that both IL-6 and sIL-6R interact exclusively with the β-propeller domain of the Vps10p-D. The fact that the tridecapeptide NT, which also targets the Vps10p-D in both SorLA and the related receptor sortilin (37, 56, 57), exhibits little or no inhibition of IL-6 and IL-6R binding obviously reflects that the tunnel of the β-propeller harbors several separate binding sites and that, unlike the propeptide which serves as a lid blocking the entrance to the tunnel, NT is simply too small to prevent other ligands from gaining access (25, 33). IL-6 and IL-6R, however, may well compete for binding to SorLA, if not because they share the same site, then due to their size. In that respect it is interesting that whereas interaction between SorLA and IL-6R appeared to exclude IL-6 from binding to either of the two (Fig. 8F), binding of IL-6 to SorLA (Fig. 9E) seemed not to interfere with IL-6:sIL-6R complex formation. This of course implies that SorLA may interact not only with IL-6 and sIL-6R but also with preformed IL-6:sIL-6R complexes in circulation.

In agreement with the *in vitro* binding experiments our findings in cell lines and wt astrocytes demonstrate that SorLA conveys rapid and efficient uptake of IL-6 and sIL-6R (and in all probability of the IL-6:sIL-6R complex). This suggests that one of SorLA's functions is to serve as a clearance receptor for these ligands and that the level of SorLA expression might impact on the level of circulating IL-6, sIL-6R, and IL-6:sIL-6R complex. This is interesting, since a number of reports have shown that elevated levels of IL-6 and sIL-6R are associated with and may partake in the development of diseases such as rheumatoid arthritis and multiple sclerosis (3, 12, 17–19). Thus, it could be speculated that SorLA (or lack of functional SorLA) also plays a role in that context.

### SorLA downregulates IL-6 *cis* signaling.

As determined by cross-linking experiments, SorLA appears to complex with the full-length transmembrane IL-6R in addition to the secreted/shed form of the receptor. SorLA has previously been reported to bind and subsequently to alter the cellular sorting of the transmembrane APP (38). We found no evidence, however, of a corresponding function in relation to IL-6R. In contrast, endocytosis, the subcellular localization, the turnover (half-life), and the shedding of the IL-6R proved unaffected by coexpression with differentially sorted SorLA constructs. Thus, in agreement with previous reports addressing IL-6R sorting and endocytosis (54, 55, 58), we find that IL-6R trafficking relies exclusively on sorting motifs within its own cytoplasmic domain. Likewise, SorLA trafficking was unaffected by IL-6R and, in a broader perspective, it is tempting to question the very concept that a receptor-A can “take over” the sorting of a receptor B (and why not vice versa?) if both carry functional sorting motifs. We have recently shown that SorLA does mediate the internalization and degradation of the CNTFR and GFRα1 (33, 34), but in the present context it is important to note that they are both glycosylphosphatidylinositol anchored and have no capacity for independent sorting.

Although SorLA appears redundant in terms of IL-6R endocytosis and sorting, our data strongly suggest that interaction between the two may modulate induction of IL-6 *cis* signaling. Thus, the response to IL-6 (in terms of pSTAT3) in cells with modest or no expression of SorLA was significantly lowered upon transfection with wt SorLA and was even further reduced when the cells were transfected with SorLAΔtail. The latter indicates that signal induction is inhibited at the cell membrane where SorLA and IL-6R interact. SorLA-mediated clearance of IL-6 can hardly account for this, since SorLAΔtail is not internalized; instead, the aforementioned observation that binding of sSorLA to IL-6R prevents interaction between IL-6 and IL-6R (Fig. 8F) might offer a plausible explanation. In other words, SorLA:IL-6R complex formation at the cell membrane may hamper IL-6's access to IL-6R, its partner in signaling. It is even conceivable that SorLA could inhibit interaction with the signal transducer gp130. Thus, the luminal domain of SorLA interacts with soluble gp130-Fc (SPR analysis [data not shown]), although we were unable to cross-link and/or coprecipitate the full-length receptors in cells.

### Soluble SorLA can act as an IL-6 carrier protein.

Whereas the data presented above suggest that membrane-bound SorLA inhibits IL-6 *cis* signaling, we find that its shed luminal part (sSorLA) may serve as a carrier protein for circulating IL-6 and IL-6:sIL-6R and preserve their capacity for *trans* signaling. This conclusion is based on the following three findings: (i) IL-6 loses biological activity upon preincubation, but (ii) its activity appears to be protected in the presence of (and binding to) sSorLA, and (iii) binding to sSorLA does not hinder or interfere with its complex formation with sIL-6R. Furthermore, it is conceivable that sSorLA, like sIL-6R (59), may prolong the plasma half-life of IL-6 by preventing delaying its degradation (functional inactivation) in blood and tissues and its elimination via the kidneys. In this context, it is interesting that membrane-bound SorLA and sSorLA may have opposite effects, since full-length SorLA tends to remove IL-6 and downregulate its activity, whereas sSorLA seems to conserve IL-6 and IL-6 bioactivity. In any event, it is likely that SorLA can influence the level of IL-6 in circulation and perhaps even the pathophysiologic conditions accompanying elevated IL-6 levels.

Finally, our present and previous studies (33, 50, 60, 61) demonstrate that Vps10p-D receptors, notably sortilin and SorLA, are new and significant participants in the functional regulation of cytokines and receptors of the IL-6 cytokine family.

## MATERIALS AND METHODS

### Reagents.

Recombinant human sIL-6R and goat anti-IL-6 were purchased from R&D Systems. The mouse anti-STAT3, rabbit anti-phosphor-STAT3 (Tyr705) (pSTAT3), and horseradish peroxidase (HRP)-linked anti-rabbit and anti-mouse antibodies were from Cell Signaling Technology, and recombinant human IL-6 and mouse anti-IL-6R (B-R6) were from Millipore. Rabbit anti-IL-6R (C-20) was obtained from Santa Cruz Biotechnology, mouse anti-cd49b was from BD Biosciences, mouse anti-LAMP-1 (H4A3) was from the Developmental Studies Hybridoma Bank, and NT was from Sigma. The murine monoclonal anti-SorLA (31) and the rabbit polyclonal anti-SorLA have previously been described (32). Secondary antibodies conjugated with Alexa Fluor dyes were all from Invitrogen.

### cDNA construct, protein expression, and purification.

Human SorLA glutathione *S*-transferase (GST)–propeptide (37) was expressed as previously described, and the hyper IL-6 was kindly provided by Stefan Rose-John (62). The pEZ-M02 expression vector comprising full-length human IL-6R cDNA (EX-A0457-M02) was purchased from GeneCopoeia, and the cDNA construct encoding human IL-6R without the cytoplasmic tail (IL-6RΔtail) was generated by introducing a stop codon at position W392 (QuikChange protocol; Stratagene) and then inserted into the pEZ-M02 vector for expression. Human cDNA constructs encoding full-length SorLA, SorLA without its cytoplasmic tail (SorLAΔtail), sSorLA, the SorLA Vps10p-D, and soluble SorCS3 (sSorCS3) were inserted into the pcDNA3.1/zeo(−) vector and expressed as described elsewhere (37, 56, 63). The recombinant expression and purification of soluble human gp130-Fc have been described elsewhere (64).

### Cell lines and transfection.

HEK293 cells were cultured in Dulbecco modified Eagle medium (DMEM) supplemented with 10% fetal bovine serum (FBS), 100 U/ml penicillin, and 100 μg/ml streptomycin. HEK293 cells were transfected using Fugene 6 (Roche) and stable clones were selected in medium containing 150 μg/ml zeocin and/or 400 μg/ml Geneticin (G418 sulfate). BA/F3-gp130, BA/F3-gp130/SorLA, BA/F3-gp130/IL-6R, and BA/F3-gp130/IL-6R/SorLA cell lines were cultured in 10% FBS-DMEM supplemented with antibiotics and 10 ng/ml IL-6 or hyper IL-6 (BA/F3-gp130 and BA/F3-gp130/SorLA). BA/F3-gp130 and BA/F3-gp130/IL-6R cells were transfected with SorLA by electroporation as previously described (60), and stably transfected clones were selected in medium containing 150 μg/ml zeocin and the appropriate cytokine.

### Immunocytochemistry.

Untransfected and SorLA-transfected HEK293 cells, and wt and SorLA ko mice astrocytes (38) were grown on cover slides and incubated with 125 nM IL-6 or 250 nM sIL-6R with or without 20 μM SorLA GST-propeptide or 20 μM NT for 30 min at 37°C. The cells were then washed in phosphate-buffered saline (PBS), fixed in formaldehyde (4%, pH 7), and permeabilized with 0.5% saponin (Sigma) prior to incubation with goat anti-IL-6 and mouse anti-SorLA or mouse anti-IL-6R and rabbit anti-SorLA. The cells were finally stained with matching conjugated secondary antibodies (Alexa Fluor). Nuclei were stained using 0.5 μg/ml bis-benzimide (Hoechst; Sigma). Staining was analyzed by confocal microscopy (LSM710 or LSM780; Carl Zeiss). Vesicles containing IL-6 or sIL-6R were quantified from images of 9 to 15 randomly selected astrocytes using the Spot detection module of Imaris 7.4.2 image analysis software (Bitplane) with a fixed intensity threshold. For inhibition of lysosomal hydrolases SorLA-transfected HEK293 cells were treated with leupeptin and pepstatin A (Sigma) as previously described (33).

HEK293 cells transfected with IL-6R, IL-6RΔtail, or either of the two in combination with SorLA were labeled with mouse anti-IL-6R and rabbit anti-SorLA for 2 h at 4°C, washed, and incubated (0 to 25 min) in warm medium (37°C). Subsequently, the cells were washed, fixed, permeabilized, and finally stained using the appropriate secondary antibodies.

### SPR.

Surface plasmon resonance (SPR) analysis was performed on a Biacore 3000 instrument (Biacore, Sweden) equipped with CM5 sensor chips activated as described previously (56). Purified soluble receptor-proteins were immobilized (at 66 to 86 fmol/mm^2^), and ligands (5 nM to 20 μM) were injected (5 μl/min) at 25°C in a 10 mM HEPES buffer (150 mM NaCl, 1.5 mM CaCl_2_, 1 mM EGTA, 0.005% Tween 20 [pH 7.4]). Binding was measured as the difference between the response obtained from the flow cell with immobilized receptor minus the response obtained when using an activated but uncoupled chip. The overall *K_d_* values were determined by BIAevaluation 4.1 software using a Langmuir 1:1 binding model and simultaneous fitting to all curves in the concentration range considered (global fitting).

### Metabolic labeling.

Metabolic labeling of HEK293 cells using [^35^S]cysteine and [^35^S]methionine (Pro-Mix; Amersham) was performed as described previously (61).

### Cross-linking and coimmunoprecipitation.

Transfected HEK293 cells were washed in PBS, followed by cross-linking for 45 min at 2 nM DTSSP (3,3′-dithiobis, sulfosuccinimidylpropionate), and reactions were stopped by the addition of 100 mM Tris (pH 7.5). After a wash in PBS, the cells were lysed at 4°C in 1% Triton X-100 lysis buffer (20 mM Tris-HCl, 10 mM EDTA [pH 8.0]) containing a proteinase inhibitor cocktail (CompleteMini; Roche). Precipitations were performed using the rabbit anti-SorLA antibody, and precipitates were subjected to immunoblot analysis using rabbit anti-SorLA and rabbit anti-IL-6R as primary antibodies and HRP-linked anti-rabbit antibody as a secondary antibody. Transfected HEK293 cells were biolabeled, washed, and lysed, and labeled receptor proteins were immunoprecipitated from the cell lysates using anti-IL-6R and analyzed by reducing SDS-PAGE and autoradiography.

### Cellular turnover of IL-6R and SorLA.

HEK293 cells, unlabeled or biolabeled in the presence of brefeldin A (36 μM), were incubated in DMEM (37°C), and at given time points the medium and/or the cell lysate (1% Triton X-100 buffer) was recovered. Labeled IL-6R in cell lysates was immunoprecipitated using mouse anti-IL-6R, and the precipitates were subjected to SDS-PAGE and autoradiography. Medium and cell lysates from unlabeled cultures were analyzed by Western blotting with rabbit anti-SorLA and rabbit anti-IL-6R as primary antibodies and HRP-linked anti-rabbit antibody as the secondary antibody.

### Subcellular fractionation.

Subcellular fractionation of HEK293 cells by discontinuous iodixanol density gradient was performed as described elsewhere (65).

### Analysis of STAT3 phosphorylation.

Ba/F3 cells were seeded in 24-well plates at 1.2 × 10^6^ cells per well, whereas murine astrocytes were seeded in 24-well plates at 1.0 × 10^5^ cells per well. The cells were then starved for 3 h in unsupplemented DMEM prior to stimulation. HEK293 cells were seeded in 24-well plates and at 50 to 80% confluence starved for 3 h in unsupplemented DMEM prior to stimulation. Stimulations were performed by incubating the starved cells with the appropriate cytokines and/or soluble receptors for 15 min (37°C). The cells were then lysed at 4°C in 1% Triton X-100 lysis buffer as described above supplemented with a phosphatase inhibitor cocktail (PhosSTOP; Roche). Supernatants containing whole-cell extracts were analyzed for protein content, and the samples were subjected to Western blot analysis with antibodies specific for STAT3, pSTAT3, SorLA, and IL-6R.

### Western blotting.

SDS-PAGE was performed using 4 to 16% gradient separation gels and NuPAGE 4 to 12% Bis-Tris protein gels (Invitrogen). For Western blotting, nitrocellulose membranes (Hybond-C; Amersham Biosciences, NJ) were blocked in TBS-T (0.01 M Tris-HCl, 0.15 M NaCl, 0.1% Tween 20 [pH 7.6]) and 5% skimmed milk powder prior to incubation with antibodies in the same buffer. Membranes were washed in TBS-T containing 0.5% skimmed milk powder.

### Statistics.

Data were evaluated either by using one-way analysis of variance (ANOVA) and Tukey's test or by using the Wilcoxon signed-rank test. For colocalization analysis, Pearson's correlation coefficient (*r*) was calculated using the Coloc 2 ImageJ plugin with default settings.

## References

[1] Scheller J, Chalaris A, Schmidt-Arras D, Rose-John S 2011 The pro- and anti-inflammatory properties of the cytokine interleukin-6. Biochim Biophys Acta 1813:878–888. doi:10.1016/j.bbamcr.2011.01.034. 21296109

[2] Rothaug M, Becker-Pauly C, Rose-John S 2016 The role of interleukin-6 signaling in nervous tissue. Biochim Biophys Acta 1863:1218–1227. doi:10.1016/j.bbamcr.2016.03.018. 27016501

[3] Erta M, Quintana A, Hidalgo J 2012 Interleukin-6, a major cytokine in the central nervous system. Int J Biol Sci 8:1254–1266. doi:10.7150/ijbs.4679. 23136554 PMC3491449

[4] Taga T, Kishimoto T 1997 Gp130 and the interleukin-6 family of cytokines. Annu Rev Immunol 15:797–819. doi:10.1146/annurev.immunol.15.1.797. 9143707

[5] Gauldie J, Richards C, Harnish D, Lansdorp P, Baumann H 1987 Interferon β2/B-cell stimulatory factor type 2 shares identity with monocyte-derived hepatocyte-stimulating factor and regulates the major acute phase protein response in liver cells. Proc Natl Acad Sci U S A 84:7251–7255. doi:10.1073/pnas.84.20.7251. 2444978 PMC299269

[6] Hirano T, Taga T, Nakano N, Yasukawa K, Kashiwamura S, Shimizu K, Nakajima K, Pyun KH, Kishimoto T 1985 Purification to homogeneity and characterization of human B-cell differentiation factor (BCDF or BSFp-2). Proc Natl Acad Sci U S A 82:5490–5494. doi:10.1073/pnas.82.16.5490. 2410927 PMC391148

[7] Kopf M, Baumann H, Freer G, Freudenberg M, Lamers M, Kishimoto T, Zinkernagel R, Bluethmann H, Kohler G 1994 Impaired immune and acute-phase responses in interleukin-6-deficient mice. Nature 368:339–342. doi:10.1038/368339a0. 8127368

[8] Lust JA, Donovan KA, Kline MP, Greipp PR, Kyle RA, Maihle NJ 1992 Isolation of an mRNA encoding a soluble form of the human interleukin-6 receptor. Cytokine 4:96–100. doi:10.1016/1043-4666(92)90043-Q. 1633265

[9] Horiuchi S, Koyanagi Y, Zhou Y, Miyamoto H, Tanaka Y, Waki M, Matsumoto A, Yamamoto M, Yamamoto N 1994 Soluble interleukin-6 receptors released from T cell or granulocyte/macrophage cell lines and human peripheral blood mononuclear cells are generated through an alternative splicing mechanism. Eur J Immunol 24:1945–1948. doi:10.1002/eji.1830240837. 8056053

[10] Mullberg J, Schooltink H, Stoyan T, Gunther M, Graeve L, Buse G, Mackiewicz A, Heinrich PC, Rose-John S 1993 The soluble interleukin-6 receptor is generated by shedding. Eur J Immunol 23:473–480. doi:10.1002/eji.1830230226. 8436181

[11] Mullberg J, Schooltink H, Stoyan T, Heinrich PC, Rose-John S 1992 Protein kinase C activity is rate limiting for shedding of the interleukin-6 receptor. Biochem Biophys Res Commun 189:794–800. doi:10.1016/0006-291X(92)92272-Y. 1335247

[12] Rose-John S, Waetzig GH, Scheller J, Grotzinger J, Seegert D 2007 The IL-6/sIL-6R complex as a novel target for therapeutic approaches. Expert Opin Ther Targets 11:613–624. doi:10.1517/14728222.11.5.613. 17465721

[13] Marz P, Heese K, Dimitriades-Schmutz B, Rose-John S, Otten U 1999 Role of interleukin-6 and soluble IL-6 receptor in region-specific induction of astrocytic differentiation and neurotrophin expression. Glia 26:191–200. 10340760 10.1002/(sici)1098-1136(199905)26:3<191::aid-glia1>3.0.co;2-#

[14] Saito M, Yoshida K, Hibi M, Taga T, Kishimoto T 1992 Molecular cloning of a murine IL-6 receptor-associated signal transducer, gp130, and its regulated expression in vivo. J Immunol 148:4066–4071. 1602143

[15] Rabe B, Chalaris A, May U, Waetzig GH, Seegert D, Williams AS, Jones SA, Rose-John S, Scheller J 2008 Transgenic blockade of interleukin 6 trans-signaling abrogates inflammation. Blood 111:1021–1028. doi:10.1182/blood-2007-07-102137. 17989316

[16] McFarland-Mancini MM, Funk HM, Paluch AM, Zhou M, Giridhar PV, Mercer CA, Kozma SC, Drew AF 2010 Differences in wound healing in mice with deficiency of IL-6 versus IL-6 receptor. J Immunol 184:7219–7228. doi:10.4049/jimmunol.0901929. 20483735

[17] Jones SA, Horiuchi S, Topley N, Yamamoto N, Fuller GM 2001 The soluble interleukin 6 receptor: mechanisms of production and implications in disease. FASEB J 15:43–58. doi:10.1096/fj.99-1003rev. 11149892

[18] Scheller J, Garbers C, Rose-John S 2013 Interleukin-6: From basic biology to selective blockade of proinflammatory activities. Semin Immunol 26:2–12. doi:10.1016/j.smim.2013.11.002. 24325804

[19] Trikha M, Corringham R, Klein B, Rossi JF 2003 Targeted anti-interleukin-6 monoclonal antibody therapy for cancer: a review of the rationale and clinical evidence. Clin Cancer Res 9:4653–4665. 14581334 PMC2929399

[20] Willnow TE, Petersen CM, Nykjaer A 2008 VPS10P-domain receptors: regulators of neuronal viability and function. Nat Rev Neurosci 9:899–909. doi:10.1038/nrg2454. 19002190

[21] Jacobsen L, Madsen P, Moestrup SK, Lund AH, Tommerup N, Nykjaer A, Sottrup-Jensen L, Gliemann J, Petersen CM 1996 Molecular characterization of a novel human hybrid-type receptor that binds the alpha2-macroglobulin receptor-associated protein. J Biol Chem 271:31379–31383. doi:10.1074/jbc.271.49.31379. 8940146

[22] Morwald S, Yamazaki H, Bujo H, Kusunoki J, Kanaki T, Seimiya K, Morisaki N, Nimpf J, Schneider WJ, Saito Y 1997 A novel mosaic protein containing LDL receptor elements is highly conserved in humans and chickens. Arterioscler Thromb Vasc Biol 17:996–1002. doi:10.1161/01.ATV.17.5.996. 9157966

[23] Kanaki T, Bujo H, Hirayama S, Ishii I, Morisaki N, Schneider WJ, Saito Y 1999 Expression of LR11, a mosaic LDL receptor family member, is markedly increased in atherosclerotic lesions. Arterioscler Thromb Vasc Biol 19:2687–2695. doi:10.1161/01.ATV.19.11.2687. 10559012

[24] Sakai S, Nakaseko C, Takeuchi M, Ohwada C, Shimizu N, Tsukamoto S, Kawaguchi T, Jiang M, Sato Y, Ebinuma H, Yokote K, Iwama A, Fukamachi I, Schneider WJ, Saito Y, Bujo H 2012 Circulating soluble LR11/SorLA levels are highly increased and ameliorated by chemotherapy in acute leukemias. Clin Chim Acta 413:1542–1548. doi:10.1016/j.cca.2012.06.025. 22750733

[25] Quistgaard EM, Madsen P, Groftehauge MK, Nissen P, Petersen CM, Thirup SS 2009 Ligands bind to sortilin in the tunnel of a ten-bladed beta-propeller domain. Nat Struct Mol Biol 16:96–98. doi:10.1038/nsmb.1543. 19122660

[26] Kitago Y, Nagae M, Nakata Z, Yagi-Utsumi M, Takagi-Niidome S, Mihara E, Nogi T, Kato K, Takagi J 2015 Structural basis for amyloidogenic peptide recognition by SorLA. Nat Struct Mol Biol 22:199–206. doi:10.1038/nsmb.2954. 25643321

[27] Fjorback AW, Seaman M, Gustafsen C, Mehmedbasic A, Gokool S, Wu C, Militz D, Schmidt V, Madsen P, Nyengaard JR, Willnow TE, Christensen EI, Mobley WB, Nykjaer A, Andersen OM 2012 Retromer binds the FANSHY sorting motif in SorLA to regulate amyloid precursor protein sorting and processing. J Neurosci 32:1467–1480. doi:10.1523/JNEUROSCI.2272-11.2012. 22279231 PMC6796259

[28] Jacobsen L, Madsen P, Nielsen MS, Geraerts WP, Gliemann J, Smit AB, Petersen CM 2002 The sorLA cytoplasmic domain interacts with GGA1 and -2 and defines minimum requirements for GGA binding. FEBS Lett 511:155–158. doi:10.1016/S0014-5793(01)03299-9. 11821067

[29] Klinger SC, Hojland A, Jain S, Kjolby M, Madsen P, Svendsen AD, Olivecrona G, Bonifacino JS, Nielsen MS 2016 Polarized trafficking of the sorting receptor SorLA in neurons and MDCK cells. FEBS J 283:2476–2493. doi:10.1111/febs.13758. 27192064 PMC7310423

[30] Taira K, Bujo H, Hirayama S, Yamazaki H, Kanaki T, Takahashi K, Ishii I, Miida T, Schneider WJ, Saito Y 2001 LR11, a mosaic LDL receptor family member, mediates the uptake of ApoE-rich lipoproteins in vitro. Arterioscler Thromb Vasc Biol 21:1501–1506. doi:10.1161/hq0901.094500. 11557679

[31] Klinger SC, Glerup S, Raarup MK, Mari MC, Nyegaard M, Koster G, Prabakaran T, Nilsson SK, Kjaergaard MM, Bakke O, Nykjaer A, Olivecrona G, Petersen CM, Nielsen MS 2011 SorLA regulates the activity of lipoprotein lipase by intracellular trafficking. J Cell Sci 124:1095–1105. doi:10.1242/jcs.072538. 21385844

[32] Nielsen MS, Gustafsen C, Madsen P, Nyengaard JR, Hermey G, Bakke O, Mari M, Schu P, Pohlmann R, Dennes A, Petersen CM 2007 Sorting by the cytoplasmic domain of the amyloid precursor protein binding receptor SorLA. Mol Cell Biol 27:6842–6851. doi:10.1128/MCB.00815-07. 17646382 PMC2099242

[33] Larsen JV, Kristensen AM, Pallesen LT, Bauer J, Vaegter CB, Nielsen MS, Madsen P, Petersen CM 2016 Cytokine-like factor 1, an essential facilitator of cardiotrophin-like cytokine:ciliary neurotrophic factor receptor alpha signaling and sorLA-mediated turnover. Mol Cell Biol 36:1272–1286. doi:10.1128/MCB.00917-15. 26858303 PMC4836274

[34] Glerup S, Lume M, Olsen D, Nyengaard JR, Vaegter CB, Gustafsen C, Christensen EI, Kjolby M, Hay-Schmidt A, Bender D, Madsen P, Saarma M, Nykjaer A, Petersen CM 2013 SorLA controls neurotrophic activity by sorting of GDNF and its receptors GFRα1 and RET. Cell Rep 3:186–199. doi:10.1016/j.celrep.2012.12.011. 23333276

[35] Westergaard UB, Sorensen ES, Hermey G, Nielsen MS, Nykjaer A, Kirkegaard K, Jacobsen C, Gliemann J, Madsen P, Petersen CM 2004 Functional organization of the sortilin Vps10p domain. J Biol Chem 279:50221–50229. doi:10.1074/jbc.M408873200. 15364913

[36] Gliemann J, Hermey G, Nykjaer A, Petersen CM, Jacobsen C, Andreasen PA 2004 The mosaic receptor sorLA/LR11 binds components of the plasminogen-activating system and platelet-derived growth factor-BB similarly to LRP1 (low-density lipoprotein receptor-related protein), but mediates slow internalization of bound ligand. Biochem J 381:203–212. doi:10.1042/BJ20040149. 15053742 PMC1133778

[37] Jacobsen L, Madsen P, Jacobsen C, Nielsen MS, Gliemann J, Petersen CM 2001 Activation and functional characterization of the mosaic receptor SorLA/LR11. J Biol Chem 276:22788–22796. doi:10.1074/jbc.M100857200. 11294867

[38] Andersen OM, Reiche J, Schmidt V, Gotthardt M, Spoelgen R, Behlke J, von Arnim CA, Breiderhoff T, Jansen P, Wu X, Bales KR, Cappai R, Masters CL, Gliemann J, Mufson EJ, Hyman BT, Paul SM, Nykjaer A, Willnow TE 2005 Neuronal sorting protein-related receptor sorLA/LR11 regulates processing of the amyloid precursor protein. Proc Natl Acad Sci U S A 102:13461–13466. doi:10.1073/pnas.0503689102. 16174740 PMC1224625

[39] Caglayan S, Takagi-Niidome S, Liao F, Carlo AS, Schmidt V, Burgert T, Kitago Y, Fuchtbauer EM, Fuchtbauer A, Holtzman DM, Takagi J, Willnow TE 2014 Lysosomal sorting of amyloid-beta by the SORLA receptor is impaired by a familial Alzheimer's disease mutation. Sci Transl Med 6:223ra220. doi:10.1126/scitranslmed.3007747. 24523320

[40] Schmidt V, Schulz N, Yan X, Schurmann A, Kempa S, Kern M, Bluher M, Poy MN, Olivecrona G, Willnow TE 2016 SORLA facilitates insulin receptor signaling in adipocytes and exacerbates obesity. J Clin Invest 126:2706–2720. doi:10.1172/JCI84708. 27322061 PMC4922706

[41] Schmidt V, Willnow TE 2016 Protein sorting gone wrong: VPS10P domain receptors in cardiovascular and metabolic diseases. Atherosclerosis 245:194–199. doi:10.1016/j.atherosclerosis.2015.11.027. 26724530

[42] Hermey G, Sjogaard SS, Petersen CM, Nykjaer A, Gliemann J 2006 Tumour necrosis factor alpha-converting enzyme mediates ectodomain shedding of Vps10p-domain receptor family members. Biochem J 395:285–293. doi:10.1042/BJ20051364. 16393139 PMC1422770

[43] Zhu Y, Bujo H, Yamazaki H, Ohwaki K, Jiang M, Hirayama S, Kanaki T, Shibasaki M, Takahashi K, Schneider WJ, Saito Y 2004 LR11, an LDL receptor gene family member, is a novel regulator of smooth muscle cell migration. Circ Res 94:752–758. doi:10.1161/01.RES.0000120862.79154.0F. 14764453

[44] Zhu Y, Bujo H, Yamazaki H, Hirayama S, Kanaki T, Takahashi K, Shibasaki M, Schneider WJ, Saito Y 2002 Enhanced expression of the LDL receptor family member LR11 increases migration of smooth muscle cells in vitro. Circulation 105:1830–1836. doi:10.1161/01.CIR.0000014413.91312.EF. 11956127

[45] Matsuo M, Ebinuma H, Fukamachi I, Jiang M, Bujo H, Saito Y 2009 Development of an immunoassay for the quantification of soluble LR11, a circulating marker of atherosclerosis. Clin Chem 55:1801–1808. doi:10.1373/clinchem.2009.127027. 19661140

[46] Ogita M, Miyauchi K, Dohi T, Tsuboi S, Miyazaki T, Yokoyama T, Yokoyama K, Shimada K, Kurata T, Jiang M, Bujo H, Daida H 2013 Increased circulating soluble LR11 in patients with acute coronary syndrome. Clin Chim Acta 415:191–194. doi:10.1016/j.cca.2012.10.047. 23127357

[47] Takahashi M, Bujo H, Shiba T, Jiang M, Maeno T, Shirai K 2012 Enhanced circulating soluble LR11 in patients with diabetic retinopathy. Am J Ophthalmol 154:187–192. doi:10.1016/j.ajo.2012.01.035. 22541650

[48] Ikeuchi T, Hirayama S, Miida T, Fukamachi I, Tokutake T, Ebinuma H, Takubo K, Kaneko H, Kasuga K, Kakita A, Takahashi H, Bujo H, Saito Y, Nishizawa M 2010 Increased levels of soluble LR11 in cerebrospinal fluid of patients with Alzheimer disease. Dement Geriatr Cogn Disord 30:28–32. doi:10.1159/000315539. 20689279

[49] Hsu MP, Frausto R, Rose-John S, Campbell IL 2015 Analysis of IL-6/gp130 family receptor expression reveals that in contrast to astroglia, microglia lack the oncostatin M receptor and functional responses to oncostatin M. Glia 63:132–141. doi:10.1002/glia.22739. 25103368

[50] Mortensen MB, Kjolby M, Gunnersen S, Larsen JV, Palmfeldt J, Falk E, Nykjaer A, Bentzon JF 2014 Targeting sortilin in immune cells reduces proinflammatory cytokines and atherosclerosis. J Clin Invest 124:5317–5322. doi:10.1172/JCI76002. 25401472 PMC4348947

[51] Dittrich E, Rose-John S, Gerhartz C, Mullberg J, Stoyan T, Yasukawa K, Heinrich PC, Graeve L 1994 Identification of a region within the cytoplasmic domain of the interleukin-6 (IL-6) signal transducer gp130 important for ligand-induced endocytosis of the IL-6 receptor. J Biol Chem 269:19014–19020. 8034658

[52] Dittrich E, Haft CR, Muys L, Heinrich PC, Graeve L 1996 A di-leucine motif and an upstream serine in the interleukin-6 (IL-6) signal transducer gp130 mediate ligand-induced endocytosis and down-regulation of the IL-6 receptor. J Biol Chem 271:5487–5494. doi:10.1074/jbc.271.10.5487. 8621406

[53] Zohlnhofer D, Graeve L, Rose-John S, Schooltink H, Dittrich E, Heinrich PC 1992 The hepatic interleukin-6 receptor. Down-regulation of the interleukin-6 binding subunit (gp80) by its ligand. FEBS Lett 306:219–222. 1321736 10.1016/0014-5793(92)81004-6

[54] Nishimoto N, Terao K, Mima T, Nakahara H, Takagi N, Kakehi T 2008 Mechanisms and pathologic significances in increase in serum interleukin-6 (IL-6) and soluble IL-6 receptor after administration of an anti-IL-6 receptor antibody, tocilizumab, in patients with rheumatoid arthritis and Castleman disease. Blood 112:3959–3964. doi:10.1182/blood-2008-05-155846. 18784373

[55] Martens AS, Bode JG, Heinrich PC, Graeve L 2000 The cytoplasmic domain of the interleukin-6 receptor gp80 mediates its basolateral sorting in polarized Madin-Darby canine kidney cells. J Cell Sci 113(Pt 20):3593–3602. 11017875 10.1242/jcs.113.20.3593

[56] Munck Petersen C, Nielsen MS, Jacobsen C, Tauris J, Jacobsen L, Gliemann J, Moestrup SK, Madsen P 1999 Propeptide cleavage conditions sortilin/neurotensin receptor-3 for ligand binding. EMBO J 18:595–604. doi:10.1093/emboj/18.3.595. 9927419 PMC1171152

[57] Mazella J, Zsurger N, Navarro V, Chabry J, Kaghad M, Caput D, Ferrara P, Vita N, Gully D, Maffrand JP, Vincent JP 1998 The 100-kDa neurotensin receptor is gp95/sortilin, a non-G-protein-coupled receptor. J Biol Chem 273:26273–26276. doi:10.1074/jbc.273.41.26273. 9756851

[58] Fujimoto K, Ida H, Hirota Y, Ishigai M, Amano J, Tanaka Y 2015 Intracellular dynamics and fate of a humanized anti-interleukin-6 receptor monoclonal antibody, tocilizumab. Mol Pharmacol 88:660–675. doi:10.1124/mol.115.099184. 26180046

[59] Peters M, Jacobs S, Ehlers M, Vollmer P, Mullberg J, Wolf E, Brem G, Meyer zum Buschenfelde KH, Rose-John S 1996 The function of the soluble interleukin 6 (IL-6) receptor in vivo: sensitization of human soluble IL-6 receptor transgenic mice towards IL-6 and prolongation of the plasma half-life of IL-6. J Exp Med 183:1399–1406. doi:10.1084/jem.183.4.1399. 8666898 PMC2192475

[60] Larsen JV, Hansen M, Moller B, Madsen P, Scheller J, Nielsen M, Petersen CM 2010 Sortilin facilitates signaling of ciliary neurotrophic factor and related helical type 1 cytokines targeting the gp130/leukemia inhibitory factor receptor beta heterodimer. Mol Cell Biol 30:4175–4187. doi:10.1128/MCB.00274-10. 20584990 PMC2937557

[61] Larsen JV, Hermey G, Sorensen ES, Prabakaran T, Christensen EI, Gliemann J, Madsen P, Petersen CM 2014 Human sorCS1 binds sortilin and hampers its cellular functions. Biochem J 457:277–288. doi:10.1042/BJ20130386. 24128306

[62] Fischer M, Goldschmitt J, Peschel C, Brakenhoff JP, Kallen KJ, Wollmer A, Grotzinger J, Rose-John S 1997 I. A bioactive designer cytokine for human hematopoietic progenitor cell expansion. Nat Biotechnol 15:142–145. 9035138 10.1038/nbt0297-142

[63] Westergaard UB, Kirkegaard K, Sorensen ES, Jacobsen C, Nielsen MS, Petersen CM, Madsen P 2005 SorCS3 does not require propeptide cleavage to bind nerve growth factor. FEBS Lett 579:1172–1176. doi:10.1016/j.febslet.2004.12.088. 15710408

[64] Jostock T, Mullberg J, Ozbek S, Atreya R, Blinn G, Voltz N, Fischer M, Neurath MF, Rose-John S 2001 Soluble gp130 is the natural inhibitor of soluble interleukin-6 receptor trans-signaling responses. Eur J Biochem 268:160–167. doi:10.1046/j.1432-1327.2001.01867.x. 11121117

[65] Chang Y, Tesco G, Jeong WJ, Lindsley L, Eckman EA, Eckman CB, Tanzi RE, Guenette SY 2003 Generation of the beta-amyloid peptide and the amyloid precursor protein C-terminal fragment gamma are potentiated by FE65L1. J Biol Chem 278:51100–51107. doi:10.1074/jbc.M309561200. 14527950

